# Omega-3 supplementation in addition to prenatal vitamins during pregnancy is associated with lower rates of preterm birth and small for gestational age

**DOI:** 10.3389/fnut.2025.1693844

**Published:** 2026-01-06

**Authors:** William Yakah, David M. Haas, William A. Grobman, Lisa D. Levine, Uma M. Reddy, Robert Silver, Claire-Marie Vacher, Ronald J. Wapner, Anna A. Penn, Morgan R. Firestein

**Affiliations:** 1 Department of Pediatrics, Columbia University Irving Medical Center, New York, NY, United States; 2 Department of Obstetrics and Gynecology, School of Medicine, Indiana University, Indianapolis, IN, United States; 3 Department of Obstetrics and Gynecology, The Ohio State University Wexner Medical Center, Columbus, OH, United States; 4 Maternal Fetal Medicine Division, University of Pennsylvania, Philadelphia, PA, United States; 5 Department of Obstetrics & Gynecology, Columbia University Irving Medical Center, New York, NY, United States; 6 Department of Obstetrics and Gynecology, University of Utah, Salt Lake City, UT, United States; 7 Child Health Institute of New Jersey, Rutgers Robert Wood Johnson Medical School, New Brunswick, NJ, United States; 8 Department of Pediatrics, Rutgers Robert Wood Johnson Medical School, New Brunswick, NJ, United States

**Keywords:** pregnancy, preterm birth, small for gestation age (SGA), prenatal vitamin, omega - 3 fatty acids, DHA, EPA

## Abstract

Omega-3 fatty acids and prenatal vitamins support fetal growth, but most studies assess omega-3 supplementation without accounting for baseline prenatal vitamin use during pregnancy. In this secondary analysis, we obtained data from the large, prospective Nulliparous Mother-to-be (nuMoM2b) cohort study of 9,461 nulliparous individuals. Participants were enrolled through eight clinical sites across the United States. We compared adverse birth outcomes between those taking additional omega-3 supplements beyond standard prenatal vitamin intake (PNV-OM) vs. prenatal vitamins alone (PNV). PNV-OM intake was associated with significantly lower rates of preterm birth (5.04 vs. 8.41%, *P* < 0.001) and SGA (2.84 vs. 4.48%, *P* = 0.004). After adjustment for demographic and clinical differences, PNV-OM use remained associated with reduced odds of preterm birth (aOR 0.64, 95% CI: 0.47–0.86, *P* = 0.004) and SGA (aOR 0.64, 95% CI: 0.42–0.95, *P* = 0.03). However, given substantial socioeconomic differences between groups and the potential for residual confounding, these findings should be interpreted with caution. Supplemental omega-3 intake during pregnancy may provide an additive benefit beyond prenatal vitamins alone, but randomized trials are needed to determine whether this relationship is causal.

## Introduction

Prenatal vitamins and omega-3 fatty acids are both essential for fetal health, supporting neurodevelopment, retinal maturation, and overall growth (1). While prenatal vitamins are widely recommended during pregnancy, they do not universally contain omega-3 fatty acids, despite evidence supporting significant benefits of omega-3s, including reducing the risk of preterm birth (2, 3). Additionally, most studies examining omega-3 supplementation during pregnancy do not account for baseline use of other prenatal vitamins (4–6), therefore, it remains unclear whether omega-3 supplementation provides additional benefits beyond those conferred by prenatal vitamins alone. Here, we examined whether omega-3 supplementation in addition to baseline prenatal vitamin intake during pregnancy is associated with reduced rates of specific adverse birth outcomes and neonatal morbidities in a cohort of nulliparous mothers enrolled in the Nulliparous Pregnancy Outcomes Study (nuMoM2b) (7).

## Materials and methods

Data were obtained through the nuMoM2b study, a multi-site prospective cohort study of 10,038 nulliparous individuals with singleton gestations recruited from eight U.S. sites from 2010 to 2013 (7). Eligible individuals were between 6^0^ and 13^6^ weeks of gestation based on an ultrasound crown-rump length measurement. Pregnant individuals were excluded if they had a prior pregnancy lasting 20 weeks' gestation or more, were younger than 13 years of age, had a history of 3 or more spontaneous abortions, or if a likely fatal fetal malformation was identified during screening. Written informed consent was obtained from all participants, and the study procedures were reviewed and approved by the Columbia University Institutional Review Board (IRB).

Participants were followed throughout the duration of their pregnancies and their self-reported use of prenatal vitamins and supplements was obtained through standardized interviews conducted at three study visits (8). At each visit, participants were asked to bring with them the labeled original packaging for the medications, vitamins, and supplements that they took (or were currently taking) during their pregnancy, and were asked to report during which trimester they started or stopped the medication. Study staff followed a standardized protocol to code the name or category of medication, initiation and cessation of each medication, and purpose of each medication. Therefore, PNV and PNV-OM intake was obtained through observational study methods rather than through randomization. Based on these data, participants were categorized into three categories of prenatal vitamin use: prenatal vitamins without documented additional omega-3 supplementation (PNV; *n* = 8,192), prenatal vitamins with additional omega-3 supplement intake (PNV-OM; *n* = 1,269; coded based on the terms, “omega-3,” “fish oil,” “DHA,” and “EPA”), or no/unknown record of prenatal vitamins intake (*n* = 577). Since product formulation details were not collected, it is possible that some prenatal vitamins taken by individuals in the PNV group contained small amounts of omega-3 fatty acids. Thus, the exposure assessed reflects intentional, supplemental omega-3 intake rather than complete absence vs. presence of omega-3 in prenatal products. Those without any documented prenatal vitamin use across all three study visits (*n* = 577) were excluded from this analysis, resulting in a total sample size of 9,461 participants. Additionally, individuals with missing data for variables included in both univariate and multivariate models were excluded from analyses.

All analyses were performed using R software (version 3.5.2, R Core team 2018a) within RStudio (Version 2024.04.1+748, RStudio, Inc., Vienna, Austria) using the “tidyverse” (9) and “CompareGroups” (10) packages. Univariable (two-sided *t*-test) and multivariable (binomial logistic regression) analyses evaluated whether the PNV-OM group differed significantly from the PNV group with regard to rates of prematurity (spontaneous or induced delivery at < 37 weeks' gestation), small-for-gestational-age birth (SGA; ≤ 5th percentile per Alexander's standard), or neonatal morbidity. Multivariable logistic regression models were constructed to estimate adjusted odds ratios (aORs) for these outcomes. Covariates were selected based on theoretical relevance and statistical significance (*P* < 0.05) in univariable analyses using two-sided *t*-tests for continuous variables and χ^2^ for categorical variables. The final models adjusted for maternal characteristics that differed statistically between groups, including advanced maternal age (≥35 years), educational attainment, poverty level, pre-pregnancy body mass index (BMI), chronic hypertension, self-reported race and ethnicity (Non-Hispanic White, Non-Hispanic Black, Asian, Hispanic, and Other/Multiracial), and the gestational timepoint when prenatal vitamin intake began. Statistical significance was defined as *P* < 0.05.

## Results

Of 9,461 participants, 8,192 (86.6%) were in the PNV group and 1,269 (13.4%) were in the PNV-OM group. Compared to those in the PNV group, PNV-OM mothers were older, more likely to be White non-Hispanic, had lower BMI, lower prevalence of chronic hypertension, higher household income, and higher educational attainment (Table 1). The PNV and PNV-OM groups did not differ significantly with regard to any other covariates that were assessed. Five preterm infants were delivered via Cesarean delivery without labor or induction. Of the remaining 755 preterm births, 93.4% occurred following spontaneous rupture of membranes. PNV-OM infants had lower rates of preterm birth (5.04 vs. 8.41%, *P* < 0.001) and SGA (2.84 vs. 4.48%, *P* = 0.004) compared to those in the PNV group. Additionally, PNV-OM infants had lower frequency of respiratory distress syndrome (RDS, 1.58 vs. 3.39%, *P* = 0.004), but similar frequencies of neonatal morbidities such as Apgar scores, bronchopulmonary dysplasia or retinopathy of prematurity. Infants in the PNV-OM group had a higher incidence of meconium aspiration syndrome (MAS) (0.95 vs. 0.51%, *P* = 0.03). Multivariable logistic regression after adjusting for maternal covariates revealed that PNV-OM intake during pregnancy was significantly associated with decreased odds of preterm birth (aOR = 0.64, 95% CI: 0.47–0.86, *P* = 0.004) and SGA (aOR = 0.64, 95% CI: 0.42–0.95, *P* = 0.03) as shown in Tables 2, 3 respectively. To assess whether the observed associations were driven by socioeconomic differences, we performed a sensitivity analysis that was restricted to participants with household incomes ≥200% of the federal poverty level (PNV *n* = 4,277, PNV-OM *n* = 1,111). Within this higher-income subset, PNV-OM use remained significantly associated with lower odds of preterm birth (aOR = 0.68; 95% CI 0.50–0.94; *P* = 0.02), but not with SGA (aOR = 0.71; 95% CI 0.46–1.07; *P* = 0.11). The associations between PNV-OM use and both preterm birth and SGA were unchanged in additional sensitivity analyses that excluded mothers aged < 18 years (PNV *n* = 7,970, PNV-OM *n* = 1,265) at delivery. Finally, given the ambiguity of reported prenatal vitamin and omega-3 intake in a small subset of participants (*n* = 192), we performed sensitivity analyses that excluded these cases. The sensitivity analyses revealed that PNV-OM use remained significantly associated with lower odds of both preterm birth (aOR = 0.60; 95% CI 0.43–0.83; *P* = 0.003) and SGA (aOR = 0.62; 95% CI 0.38–.94; *P* = 0.03).

**Table 1:** Maternal and neonatal demographic and clinical characteristics.

**Variable**	**PNV only (*N* = 8,192)**	**PNV-OM (*N* = 1,269)**	**T-statistic or χ^2a^**	***P*-value**
**Maternal characteristics**				
Maternal age	26.4 ± 5.61	30.4 ± 4.52	−24.98	**< 0.0001**
**Age category**			165.01	**< 0.0001**
13–17	222 (2.71%)	4 (0.32%)		
18–34	7,330 (89.5%)	1,037 (81.7%)		
≥35	640 (7.81%)	228 (18.0%)		
BMI	26.6 ± 6.48	25.2 ± 5.17	8.58	**< 0.0001**
**Self-reported race**			291.05	**< 0.0001**
Non-Hispanic White	4,708 (57.5%)	999 (78.7%)		
Non-Hispanic Black	1,279 (15.6%)	40 (3.15%)		
Hispanic	1,478 (18.0%)	100 (7.88%)		
Asian	305 (3.72%)	78 (6.15%)		
Other	422 (5.15%)	52 (4.10%)		
**Income level**			315.68	**< 0.0001**
≤ 100% federal poverty level	1,186 (14.5%)	29 (2.3%)		
100–200% federal poverty level	1,015 (12.4%)	81 (6.4%)		
≥200% federal poverty level	4,277 (52.2%)	1,111 (87.5%)		
Unknown/not reported	1,714 (20.9%)	48 (3.8%)		
**Education**			490.27	**< 0.0001**
≤ High School graduate	1,796 (21.9%)	51 (4.02%)		
Some college or Assoc/Tech degree	2,576 (31.4%)	213 (16.8%)		
Completed college or more	3,813 (46.5%)	1,005 (79.2%)		
Unknown/not reported	7 (0.09%)	0 (0.0%)		
**When prenatal vitamins were started**			475.57	**< 0.0001**
Before pregnancy	3,143 (38.4%)	621 (48.9%)		
During 1st trimester	4,567 (55.7%)	456 (35.9%)		
During 2nd trimester	188 (2.29%)	89 (7.01%)		
During 3rd trimester	38 (0.46%)	77 (6.07%)		
Unknown/not reported	256 (3.13%)	26 (2.05%)		
**Diabetes**			5.00	0.082
None	7,532 (91.9%)	1,196 (94.2%)		
Pre-gestational diabetes	136 (1.66%)	13 (1.02%)		
Gestational diabetes	342 (4.17%)	43 (3.39%)		
Unknown/not reported	182 (2.22%)	17 (1.34%)		
Chronic hypertension	219 (2.67%)	19 (1.50%)	6.01	**0.014**
Gestational-Onset hypertension	1,111 (13.6%)	186 (14.7%)	2.60	0.395
**Neonatal characteristics**				
Preterm birth	689 (8.41%)	64 (5.04%)	17.38	**< 0.0001**
**Premature rupture of membranes (PROM)**			5.71	0.477
None	7,503 (91.6%)	1,205 (95.0%)		
Spontaneous	639 (7.80%)	59 (4.65%)		
Induced	46 (0.56%)	4 (0.32%)		
Cesarean without labor or induction	4 (0.05%)	1 (0.08%)		
Small for gestational age (SGA)	367 (4.48%)	36 (2.84%)	7.31	**0.004**
Gestational age (weeks)	38.4 ± 3.46	38.9 ± 2.70	−5.81	**< 0.0001**
Birthweight (g)	3,254 ± 584	3,351 ± 517	−5.99	**< 0.0001**
Birth length (cm)	50.5 ± 3.74	51.2 ± 3.41	−6.04	**< 0.0001**
Birth head circumference (cm)	34.0 ± 2.03	34.3 ± 1.77	−5.22	**< 0.0001**
1-min Apgar	7.80 ± 1.64	7.78 ± 1.70	0.52	0.600
5-min Apgar	8.78 ± 0.79	8.80 ± 0.68	−1.03	0.302
Respiratory distress syndrome	278 (3.39%)	20 (1.58%)	8.28	**0.004**
Bronchopulmonary dysplasia	14 (0.17%)	3 (0.24%)	0.13	0.443
Retinopathy of prematurity	30 (0.37%)	5 (0.39%)	0.03	0.598
Meconium aspiration syndrome	42 (0.51%)	12 (0.95%)	4.75	**0.029**
Persistent pulmonary hypertension	17 (0.21%)	2 (0.16%)	< 0.01	1

Maternal and neonatal characteristics were expressed as mean ± standard deviation for continuous variables, and proportion (%) for categorical variables. Gestational-Onset Hypertension includes new onset antepartum hypertension, mild preeclampsia, severe preeclampsia, superimposed preeclampsia, and eclampsia. Percentages are based on non-missing data for each variable. Denominators for percentages may vary due to missing data. ^a^Comparisons for continious variables conducted using the t-test and comparisons for categorical variables conducted using χ^2^. PNV, Prenatal vitamins without documented additional omega-3 supplementation; PNV-OM, Prenatal vitamins with additional omega-3 intake. P-values in bold font highlight statistically significant differences.

**Table 2:** Multivariable analyses between Prenatal Vitamins (PV) + Omega-3 supplements (PVOM) intake compared to PV only during pregnancy, on birth outcomes.

**Variable**	**Risk of prematurity**			
	**Unadjusted OR [95% CI]**	***P*** **-value**	**Adjusted OR [95% CI]**	***P*** **-value**
PNV-OM vs PNV only	0.57 [0.43, 0.74]	**< 0.001**	0.64 [0.47, 0.86]	**0.004**
Advanced maternal age	1.39 [1.10, 1.75]	**0.005**	1.56 [1.19, 2.05]	**0.001**
Pre-pregnancy BMI	0.98 [0.97, 0.99]	**0.004**	1.02 [1.01, 1.04]	**< 0.001**
Chronic hypertension	3.44 [2.49, 4.70]	**< 0.001**	3.06 [2.07, 4.44]	**< 0.001**
**Self-reported race**				
Non-Hispanic White	Ref.		Ref.	
Non-Hispanic Black	1.58 [1.29, 1.92]	**< 0.001**	1.09 [0.81, 1.45]	0.560
Hispanic	0.97 [0.78, 1.19]	0.746	0.76 [0.56, 1.02]	0.074
Asian	0.78 [0.49, 1.18]	0.265	0.91 [0.55, 1.44]	0.713
Other	1.22 [0.87, 1.69]	0.225	1.02 [0.67, 1.51]	0.912
**Education**				
≤ High school graduate	Ref.		Ref.	
Some college or Assoc/Tech degree	0.84 [0.70, 1.03]	0.094	0.85 [0.65, 1.13]	0.255
Completed college or more	0.56 [0.46, 0.68]	**< 0.001**	0.57 [0.42, 0.79]	**< 0.001**
**Income level**				
≤ 100% federal poverty level	Ref.		Ref.	
100%−200% federal poverty level	0.87 [0.65, 1.16]	0.341	1.00 [0.73, 1.37]	0.996
≥200% federal poverty level	0.70 [0.57, 0.88]	**0.002**	1.03 [0.77, 1.39]	0.837
**When prenatal vitamins were started**				
Before pregnancy	Ref.		Ref.	
During 1st trimester	1.27 [1.08, 1.49]	**0.003**	0.94 [0.77, 1.15]	0.540
During 2nd trimester	1.00 [0.60, 1.56]	0.986	0.86 [0.44, 1.53]	0.630
During 3rd trimester	0.94 [0.42, 1.84]	0.878	1.17 [0.48, 2.44]	0.702

P-values in bold font highlight statistically significant differences.

**Table 3:** Multivariable analyses between Prenatal Vitamins (PV) + Omega-3 supplements (PVOM) intake compared to PV only during pregnancy, on small for gestational age (SGA).

**Variable**	**Risk of small-for-gestational age (SGA)**			
	**Unadjusted OR [95% CI]**	***P*** **-value**	**Adjusted OR [95% CI]**	***P*** **-value**
PNV-OM vs PNV only	0.61 [0.43, 0.86]	**0.006**	0.64 [0.42, 0.95]	**0.033**
Advanced maternal age	1.16 [0.83, 1.59]	0.367	1.33 [0.89, 1.93]	0.154
Pre-pregnancy BMI	0.97 [0.96, 1.00]	**0.016**	0.96 [0.94, 0.98]	**< 0.001**
Chronic hypertension	1.58 [0.91, 2.57]	0.080	2.85 [1.55, 4.92]	**< 0.001**
**Self-reported race**				
Non-Hispanic White	Ref.		Ref.	
Non-Hispanic Black	2.06 [1.57, 2.66]	**< 0.001**	1.53 [1.02, 2.25]	**0.034**
Hispanic	1.49 [1.13, 1.94]	**0.004**	1.21 [0.82, 1.74]	0.315
Asian	1.91 [1.21, 2.90]	**0.004**	1.80 [1.06, 2.89]	**0.021**
Other	1.38 [0.86, 2.12]	0.161	1.12 [0.60, 1.89]	0.705
**Education**				
≤ High school graduate	Ref.		Ref.	
Some college or Assoc/Tech degree	0.80 [0.62, 1.03]	0.087	0.83 [0.57, 1.22]	0.337
Completed college or more	0.53 [0.41, 0.68]	**< 0.001**	0.55 [0.36, 0.85]	**0.006**
**Income level**				
≤ 100% federal poverty level	Ref.		Ref.	
100–200% federal poverty level	0.68 [0.45, 1.01]	0.058	0.78 [0.50, 1.20]	0.263
≥200% federal poverty level	0.66 [0.50, 0.89]	**0.005**	1.02 [0.69, 1.53]	0.912
**When prenatal vitamins were started**				
Before pregnancy	Ref.		Ref.	
During 1st trimester	1.32 [1.07, 1.63]	**0.011**	0.98 [0.74, 1.28]	0.859
During 2nd trimester	1.06 [0.53, 1.89]	0.857	0.84 [0.32, 1.82]	0.694
During 3rd trimester	0.89 [0.27, 2.15]	0.819	1.19 [0.35, 3.01]	0.746

P-values in bold font highlight statistically significant differences.

## Discussion

In this study, we investigated whether additional use of omega-3 supplements with prenatal vitamins would be associated with a lower rate of adverse birth outcomes and neonatal morbidities, compared to the use of prenatal vitamins alone. We found that omega-3 supplementation in addition to prenatal vitamin use (PNV-OM) was associated with a significantly lower risk of preterm birth and SGA compared to use of prenatal vitamins without omega-3 (PNV), with the odds of preterm birth and SGA reduced by 36% and 35%, respectively. Although the observed reduction in preterm birth exceeds effect sizes reported in prior literature, such as the ~11% reduction in meta-analytic evidence (2), these comparisons should be interpreted with caution, as observational designs may be more strongly influenced by unmeasured confounding and selection bias than randomized trials. We also observed a higher incidence of meconium aspiration syndrome (MAS) among infants in the PNV-OM group; however, given the small number of cases and the absence of a known biological mechanism linking additional omega-3 supplementation to increased MAS risk, this finding may reflect chance, residual confounding, type I error, or differences in clinical care or documentation practices between groups.

Women in the PNV-OM group were substantially more socioeconomically and demographically advantaged than those in the PNV group—including higher educational attainment, higher household income, older age, and a greater proportion identifying as non-Hispanic White. These factors are strongly associated with improved pregnancy outcomes independent of nutritional supplement use (11). Although our models adjusted for these variables, the magnitude of imbalance between groups makes residual confounding highly likely. Individuals who choose to take both prenatal vitamins and omega-3 supplements are also likely to engage in other unmeasured health-promoting behaviors (e.g., earlier and more consistent prenatal care, greater health literacy, or superior diet quality) that we could not account for, any of which may have contributed to the observed associations. This interpretation is supported by our sensitivity analysis restricted to participants with higher incomes, in which the association with SGA was attenuated and no longer statistically significant—underscoring that socioeconomic advantage may account for at least part of the observed effect. Thus, while our findings suggest a potential benefit of omega-3 supplementation when used in addition to prenatal vitamins, the results should be interpreted with caution in light of these potential limitations, as socioeconomic and behavioral confounding may explain much of the association.

To the extent that our findings may reflect a causal relationship, several biologically plausible mechanisms may underlie the apparent benefits of omega-3 fatty acids on pregnancy outcomes. Omega-3 fatty acids may contribute to improved outcomes through multiple pathways, including attenuation of inflammatory processes, as reported in supplementation trials (12), and enhancement of placental function via optimized transfer of long-chain polyunsaturated fatty acids to the fetus, which are essential for brain and lung development (13). These mechanisms may partly explain the observed reductions in preterm birth and SGA, as well as the lower rates of RDS in the PNV-OM group; however, direct evidence linking omega-3 supplementation to improved placental function, fetal lung maturation or neonatal respiratory outcomes remains limited and warrants further investigation.

In summary, the present study contributes to the growing evidence that omega-3 fatty acid supplementation in addition to prenatal vitamins during pregnancy is associated with a reduced risk of preterm birth and SGA; however, given the potential of residual confounding in observational studies, randomized controlled trials are needed to determine whether omega-3 supplementation can robustly promote healthy pregnancy outcomes.

## Data Availability

Publicly available datasets were analyzed in this study. This data can be found at: NICHD DASH.
